# An optimal strategy to solve the Prisoner’s Dilemma

**DOI:** 10.1038/s41598-018-20426-w

**Published:** 2018-01-31

**Authors:** Alessandro Bravetti, Pablo Padilla

**Affiliations:** 1 Instituto de Investigaciones en Matemáticas Aplicadas y en Sistemas, Universidad Nacional Autónoma de México, México City, 04510 Mexico; 2 Fitzwilliam College, University of Cambridge, Storey’s Way, CB3 ODG UK

## Abstract

Cooperation is a central mechanism for evolution. It consists of an individual paying a cost in order to benefit another individual. However, natural selection describes individuals as being selfish and in competition among themselves. Therefore explaining the origin of cooperation within the context of natural selection is a problem that has been puzzling researchers for a long time. In the paradigmatic case of the Prisoner’s Dilemma (PD), several schemes for the evolution of cooperation have been proposed. Here we introduce an extension of the Replicator Equation (RE), called the Optimal Replicator Equation (ORE), motivated by the fact that evolution acts not only at the level of individuals of a population, but also among competing populations, and we show that this new model for natural selection directly leads to a simple and natural rule for the emergence of cooperation in the most basic version of the PD. Contrary to common belief, our results reveal that cooperation can emerge among selfish individuals because of selfishness itself: if the final reward for being part of a society is sufficiently appealing, players spontaneously decide to cooperate.

## Introduction

Cooperation is so important that it has been suggested as a fundamental principle of evolution, besides reproduction, mutation and selection^1–13^. However, the situation regarding the underlying mechanisms responsible for the emergence of cooperation among individuals who try to maximize their own fitness and who are competing among each other is not clear. To try to solve such complicated puzzle, several mechanisms have been proposed during the last decades, including kin selection^1,6^, direct reciprocity^2,3,14^, indirect reciprocity^15,16^, network reciprocity^17,18^, group selection^9,19^, green beards^5,20^, optional participation^21,22^, punishment and reward^23,24^, pre-commitments^25–27^ and others^28–31^. All these situations reflect some specific important aspects of real social and biological interactions. However, none of them can really provide a solution to the most basic version of the paradigmatic example of the Prisoner’s Dilemma (PD): here one imagines that two people that are suspected of having committed a joint crime are caught by the police and confined into different rooms, without the possibility to communicate. Each of them is offered the possibility to confess the crime and defect his partner in exchange for a reduced sentence. If only one defects, the other will get the full sentence. If they defect, both will have the sentence reduced. If they cooperate between themselves and do not confess, then they will immediately be freed. The situation can be exemplified in the following payoff matrix

1 $$\begin{array}{cc} & \begin{array}{cc}C & D\end{array}\\ \begin{array}{c}C\\ D\end{array} & (\begin{array}{ll}R & S\\ T & P\end{array})\end{array},$$

where C stands for “cooperation”, D for “defection”, and the entries denote the payoff for the row player. Thus, if they both cooperate, they get *R* points each (the “reward” for cooperation). If only one cooperates, then he gets *S* points (the “sucker’s” payoff), while the defector gets *T* (the “temptation” to defect). If they both defect, they get *P* points each (the “punishment” for defection).

As it is well-known, in the PD the payoffs satisfy *T* > *R* > *P* > *S*. In this situation the best option for each player is to defect, no matter what the other player does^32^. Thus both players defect and get *P* points each. However, this is less than the *R* points they would get if they had collaborated. This is the essence of the PD: mutual cooperation leads to a higher payoff than mutual defection, but it is not a “safe” strategy, exposing the player to exploitation by a defector, and therefore each player, in an attempt to maximize his own payoff, chooses to defect. Thus apparently there is no room for cooperation between such rational agents.

We remark also that in this one-shot formulation of the PD, the game is not repeated (and thus direct and indirect reciprocity do not apply), there is no link between the two prisoners (neither a genetic one as in kin selection, nor a “social” one, as in the cases of network reciprocity and group selection), nor is there a special tag that the prisoners can use (thus ruling out green beards and tag-based donation) and finally, the two prisoners cannot decide whether to play or not (and hence optional participation cannot be used). Therefore our best candidate mechanisms for the emergence of a cooperative strategy between the two prisoners do not apply in this paradigmatic example and we are left with the fundamental question: is there any other reason that can lead the two individuals to cooperate in this case?

A related unsatisfactory aspect in the formulation of evolutionary biology in terms of replicator-type dynamics with a fixed fitness landscape is the lack of adaptability: since the payoff structure is set from the beginning, e.g. (1) for the PD, and it completely determines the fitness of each strategy, the model is not flexible enough in order to incorporate changes in the payoffs which might happen over time. To overcome such difficulty, dynamical models for the coevolution of the existing species and their fitness landscape have been proposed^33–36^. This subject is also fundamentally linked to the fact that evolution is similar to an optimization process: a population evolves in order to adapt as much as possible to some external conditions (which in turn may change in time). Some works from different perspectives have been recently proposed in this context^37–39^. In particular, in^38^ it has been argued that living systems adapt to the environment by performing an optimal control.

The aim of this work is to present a dynamical mechanism based on optimal control theory that has two main features: on the one side it generalizes the standard replicator-type dynamics to an evolution which can automatically adapt to changes in the fitness landscape by exerting a dynamical control on the fitness itself and on the other side it provides a simple and natural rule for the emergence of cooperation in the basic formulation of the PD presented above. Remarkably, this rule seems quite reasonable for the two prisoners as well as in biological and social interactions.

## Methods

To analyze the PD from a dynamical point of view, we need to switch the perspective from game theory to evolutionary dynamics. Here the different strategies correspond to different types of individuals in a population competing by natural selection and the payoffs correspond to each type’s ability to reproduce, which is called the *fitness*. For simplicity, we consider a population with only two possible types (this is all we need in order to address the PD; including more types is straightforward in principle, but the calculations get more involved very quickly). The fundamental equation of evolutionary dynamics is the Replicator Equation (RE)

2 $${\dot{x}}_{a}={x}_{a}({f}_{a}({\bf{x}})-\langle {\bf{f}}\rangle )\,\quad a=1,2,$$

where *x*_*a*_ is the relative abundance (frequency) of individuals of type *a*, *f*_*a*_(**x**) is the (frequency-dependent) fitness of type *a*, $\langle {\bf{f}}\,\rangle ={x}_{1}\,{f}_{1}+{x}_{2}\,{f}_{2}$ is the average fitness of the population and the overdot denotes time derivative.

For the PD, there are only two possible strategies: C or D. Thus we can label the frequency of the population adopting each strategy as *x*_*C*_ and *x*_*D*_ respectively. Moreover, the fitness of each strategy is obtained from the payoff matrix (1) by combining each payoff with the probability that the opponent chooses the corresponding strategy, thus obtaining

3 $${f}_{C}({\bf{x}})=R{x}_{C}+S{x}_{D}\,,\quad {f}_{D}({\bf{x}})=T{x}_{C}+P{x}_{D}.$$

Without loss of generality, we assume *S* = 0. Using the fact that *x*_*C*_ + *x*_*D*_ = 1, after some algebra we can rewrite the RE and the fitnesses in terms of *x*_*C*_ only, obtaining the following evolution equation for the frequency of cooperators

4 $${\dot{x}}_{C}=-{x}_{C}\mathrm{(1}-{x}_{C})[(T-R){x}_{C}+P\mathrm{(1}-{x}_{C})],$$

**Figure 1:**
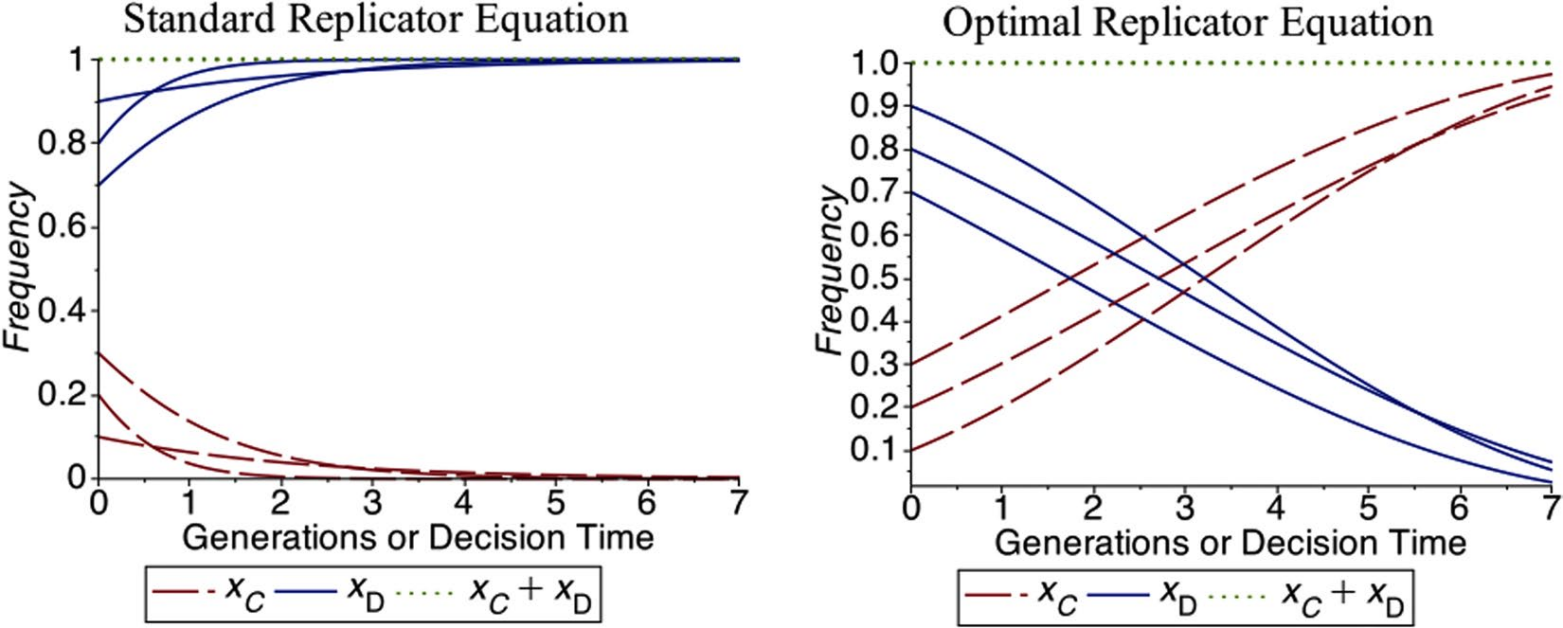
Evolution of cooperators (C) and defectors (D) in the PD. On the left panel we present the evolution according to the standard Replicator Equation, while the right panel is the evolution of the same system according to the Optimal Replicator Equation introduced here. The payoffs and the initial conditions used in these examples are reported in Table 1. While the standard RE predicts that cooperators disappear during evolution, the ORE shows the emergence of cooperation.

From which it is easy to deduce that there are only two fixed points *x*_*C*_ = 0,1, and that only *x*_*C*_ = 0 is stable, that is, cooperators are dominated by defectors (cf. Fig. 1).

**Table 1:** Models used for the graphics.

	*T*	*R*	*P*	*S*	${{\boldsymbol{x}}}_{{\boldsymbol{C}}}^{{\bf{0}}}$
Model 1	5	4	1	0	0.3
Model 2	4	3	2	0	0.2
Model 3	4	3.5	0.5	0.5	0.1

**Figure 2:**
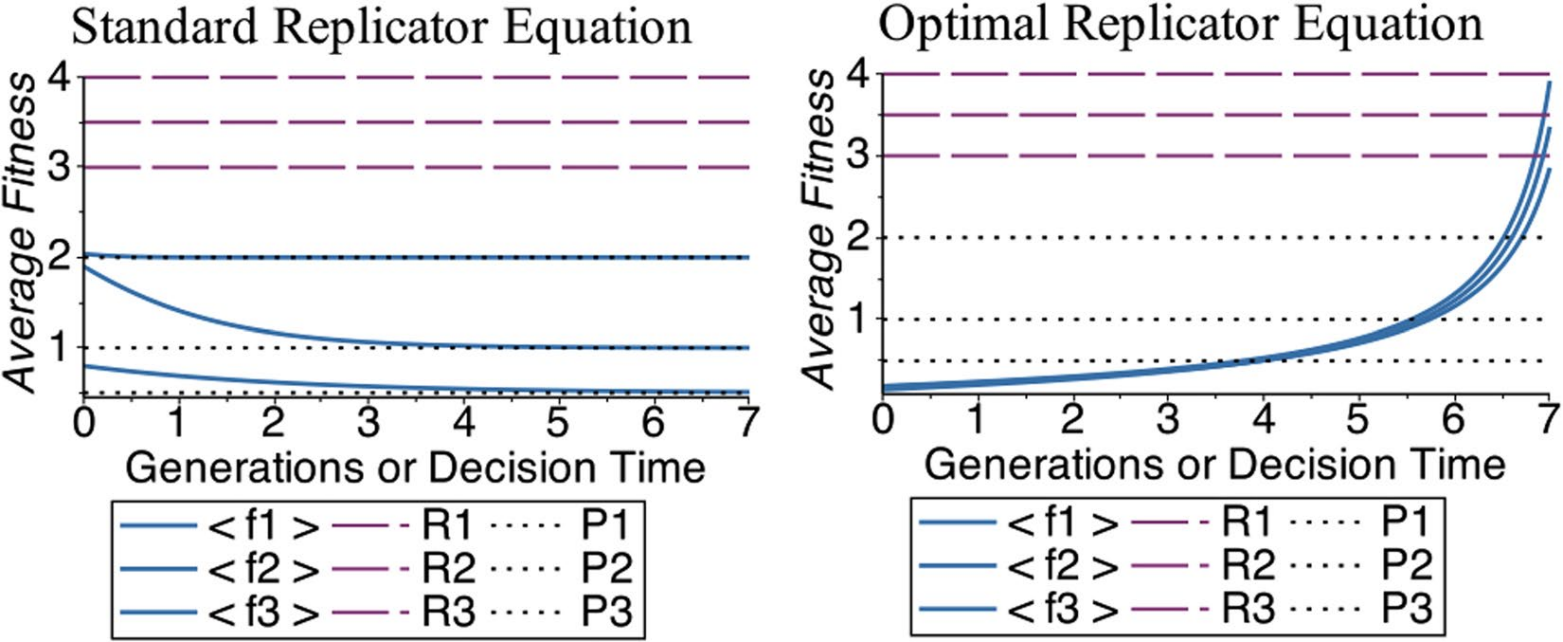
Evolution of the average fitness of the population in the PD. On the left panel we present the evolution according to the standard Replicator Equation, while the right panel is the evolution of the same system according to the Optimal Replicator Equation introduced here. The payoffs and the initial conditions used in these examples are reported in Table 1. While using the standard RE the average fitness of the population decreases and approaches the payoff for mutual defection (*P*), in the system governed by the ORE the average fitness increases and approaches the payoff for mutual cooperation (*R*).

We report the values of the payoffs and of the initial conditions used in the different models given in Figs 1 and 2.

Thus both game theory and evolutionary dynamics agree on the fact that the best strategy in the PD is to defect. Remarkably, the standard RE always favours defectors over cooperators whenever *T* > *R* > *P* > *S*, independently of their relative values. Notwithstanding, one would expect that in real situations there should be a difference between e.g. the case *T* = 100, *R* = 10, *P* = 9, *S* = 0 and the case *T* = 5, *R* = 4, *P* = 1, *S* = 0. Notice also that in this classical treatment of the PD, the average fitness of the population decreases over time (see Fig. 2), meaning that the optimal strategy (defection) is optimal only with respect to the local payoff of each player, but it is not optimal with respect to the global payoff of the entire population. This is again the essence of the PD: by trying to maximize only their individual fitness, the prisoners do not achieve the best available fitness. Interestingly, this is unstable whenever the population is not isolated, but it is under evolutionary pressure by some other population, meaning that whenever selection acts both at the level of single individuals in a given population and at the level of competing populations, a strategy considering only the former aspect is destined to be suppressed by one considering both features (this is the main reason why group selection achieves cooperation^9,19^).

Let us now use this observation in order to construct a mechanism that boosts the emergence of collaboration among competing individuals. Motivated by the fact that being part of a society provides in many situations a concrete evolutionary advantage for each organism, we assume that natural evolution acts by two mechanisms: on the one side it selects those individuals inside a population with higher fitness at any given time, and on the other side it selects those populations with the higher final average fitness (we can see this process as due to the existence of two time scales, a faster one, in which selection operates at the level of individuals in a population, and a slower scale, in which selection operates among populations). We require the evolution in the faster scale to be dictated locally in time by the standard RE. Finally, in order to obtain a model sensitive to changes in the environment, we suppose that the fitness of each strategy can be regulated in response to the present state of the population (this is similar to the coevolution mechanisms proposed e.g. in refs^33,34^).

The above assumptions can be translated into the mathematical problem of maximizing a functional which consists of two terms (one corresponding to selection at the level of individuals, and one corresponding to selection at the level of populations) subject to a dynamical constraint set by the standard RE. In order to find the best strategy to realize this task, we need to solve a maximization problem (similar ideas have been put forward in^37–39^). This is the arena of Optimal Control Theory (OCT)^40–43^. By applying OCT we obtain (see Supplementary Information) an enlarged system of equations that extends the RE (2) and describes the dynamical equations for the coevolution of the frequencies **x** in the RE and the fitnesses **f**. For *a* = *C*, *D*, as in the case of the PD, this system reads

5 $${f}_{a}=\frac{1}{2}\,{p}_{a},$$

6 $${\dot{x}}_{a}=\frac{{x}_{a}}{2}({p}_{a}-\langle {\bf{p}}\rangle ),$$

7 $${\dot{p}}_{a}=\frac{{p}_{a}}{2}(\langle {\bf{p}}\rangle -\frac{{p}_{a}}{2})\mathrm{.}$$

The additional variables *p*_*C*_ and *p*_*D*_ are usually called the *co-states* (see Supplementary Information). We call the system (5)–(7), together with the initial conditions ${x}_{a}\mathrm{(0)}={x}_{a}^{0}$ and the terminal conditions

8 $${p}_{a}(\tau )=\frac{\partial g({\bf{x}}(\tau ))}{\partial {x}_{a}},\quad \quad g({\bf{x}})\,:=\langle {\bf{f}}\rangle $$

the *Optimal Replicator Equation* (ORE). In the following we show that the ORE leads directly to a simple rule for the emergence of cooperation in the PD.

## Results

Let us consider now the PD in terms of the ORE. As usual, we use *x*_*C*_ + *x*_*D*_ = 1 to simplify (6),(7) by eliminating the equation for *x*_*D*_. Indeed, one can use ${\dot{x}}_{D}=-{\dot{x}}_{C}$ to rewrite the two equations in (6) as a single equation for *x*_*C*_, which reads

9 $${\dot{x}}_{C}={x}_{C}\mathrm{(1}-{x}_{C})\frac{{p}_{C}-{p}_{D}}{2}.$$

Thus we see that we have obtained already a simple condition for the emergence of cooperation, that is, ${p}_{C}-{p}_{D} > 0$. Since by the optimal control strategy (5), *p*_*C*_ and *p*_*D*_ correspond to the fitness of cooperators and defectors respectively, the above condition seems almost trivial at first sight: cooperation emerges whenever *f*_*C*_ > *f*_*D*_. Nevertheless, this condition is not trivial in this case, at least for two reasons: firstly, it is obtained as the result of an optimal strategy; secondly, and most importantly, because the two variables *p*_*C*_ and *p*_*D*_ are *dynamical*, with dynamical equations (7) and terminal conditions determined by the choice of the final average fitness according to (8). This aspect is crucial in the solution of the PD.

Let us return to the case of our two prisoners, with payoff matrix as in (1). Now we consider the payoffs in (1) as a *final* payoff given at time *t* = *τ* and use the ORE with the final fitness for each strategy given by (3). As before, we assume *S* = 0, thus the final average fitness is

10 $$g({\bf{x}}(\tau ))=R{x}_{C}{(\tau )}^{2}+T{x}_{C}(\tau ){x}_{D}(\tau )+P{x}_{D}{(\tau )}^{2},$$

and the terminal conditions (8) read

11 $${p}_{C}(\tau )=(2R-T){x}_{C}(\tau )+T,$$

12 $${p}_{D}(\tau )=(T-2P){x}_{C}(\tau )+2P.$$

the system of equations (7) and (9), together with the initial conditions ${x}_{C}\mathrm{(0)}={x}_{C}^{0}$ and ${x}_{D}\mathrm{(0})=1-{x}_{C}^{0}$ and the terminal conditions (11),(12) is our dynamical model for the PD. Using (9) and (11),(12) one can prove that for

13 $$2P\le T\le 2R,$$

the right hand side of (9) at *t* = *τ* is always greater than or equal to zero, with equality only for *x*_*C*_ = 0, 1. This means that this evolution admits only two equilibria, namely *x*_*C*_ = 0,1 and that only *x*_*C*_ = 1 is asymptotically stable. Therefore (13) is the condition for the emergence of cooperation in the PD using the ORE. Typical numerical solutions are given in Fig. 1. As we see, contrary to the standard RE, according to the ORE cooperators take over the population.

Another difference with respect to the standard RE is that the average fitness of the population *increases* with the ORE: while the standard evolution dictated by the RE predicts that the average fitness of the population decreases, approaching the payoff of mutual defection (see Fig. 2), the ORE predicts that selfish individuals cooperate in order to maximize the final average fitness of the population, because this entails an advantage for themselves.

The reason for the cooperative behavior in the ORE is simple: using the ORE we have extended the RE to a dynamics which considers the important evolutionary advantage for each individual deriving from being in a population that has a large final average fitness. Knowing that in case of collaboration they will be able to share an important final payoff, naturally induces the two prisoners to collaborate. Interestingly, collaboration only appears whenever *R* is “high enough”, that is, whenever 2*R* ≥ *T*, and also whenever *P* is “low enough”, that is, whenever 2*P* ≥ *T*. In both cases the prisoners do not find advantageous to defect, and prefer to take the risk to be exposed to exploitation rather than taking a lower payoff. They consider that in such cases the risk is worth the price (cf.^44^).

In biology we can safely assume that in many situations being part of a society guarantees a much higher probability of survival, for instance because it provides better strategies for the collection of food, or for reproduction, or for defense against predators. In this biological setting, we argue that the effect of cooperation can only be assessed at the level of the average fitness of the whole population. So, even if a tendency to cooperate might be inherited, its evolutionary advantage can only be evaluated collectively a posteriori and therefore cannot be included in a local term for the RE, but rather as a final condition. A striking example is provided by bees: in bees society, usually only queens can reproduce. Therefore an evolution based only on the ability to reproduce would lead very quickly to the disappearance of any worker bee. However, workers play an important role for finding food and defending the hive. Cooperation between queens and workers means that the former guarantee reproduction for the latter, while the latter work for the former. This leads to a huge final reward, that is, the conservation of the species after each generation.

Finally, let us stress that while in the case of the PD the two prisoners can be aware of the final reward for collaboration and therefore they can (consciously) decide to cooperate under the appropriate conditions, in a biological setting we can no longer give a similar interpretation. However, as it is usual in evolutionary dynamics, we suppose that the different individuals in the population inherit the possible traits randomly and that evolution favours only those individuals with the best traits, so that the optimization process is a consequence of natural selection and not a conscious decision in such case.

## Discussion

To summarize, we have proposed a modified version of the Replicator Equation (RE), the equation governing natural selection, called the Optimal Replicator Equation (ORE), which stems from the assumption that evolution is an optimization process that on the one side selects at any given time those individuals with higher fitness and on the other side favours those populations with higher average fitness. The main motivations for the introduction of such model are the facts that the standard RE cannot account for selection on the two levels of individuals and populations and that it fails to reproduce observed situations, such as the emergence of cooperation in the Prisoner’s Dilemma. Interestingly, by implementing our model (which by definition takes into account the two levels of selection among individuals and populations) to the case of the PD, we have shown that the corresponding dynamics naturally favours cooperation in the case of the basic Prisoner’s Dilemma under some reasonable conditions (cf. (^13^)). Our results thus open the door for an investigation of evolution and social dilemmas in terms of optimization by using the reproduction coefficients – i.e. the fitness – as control parameters. In particular, it would be interesting for future work to compare the condition for the emergence of cooperation obtained here with data from various experiments on the PD and with conditions derived from similar models^44–46^. Moreover, one can enlarge the study of the optimal strategies deriving from the ORE by considering different social dilemmas beside the PD. We expect to find results on the emergence of cooperation similar to the ones presented here, thus enforcing the idea that the ORE can be a good dynamical model for explaining the emergence of cooperation in a competitive framework.

## Electronic supplementary material

Supplementary Information
