# Supplementary material for: An optimal strategy to solve the Prisoner’s Dilemma
Source: Sci Rep. 2018 Jan 31;8:1948. doi: 10.1038/s41598-018-20426-w (PMC5792647; doi:10.1038/s41598-018-20426-w)
Supplement: Supplementary file 1 — Supplementary Information [file 41598_2018_20426_MOESM1_ESM.pdf]

Supplementary Information for "An optimal strategy to solve the Prisoner's Dilemma"

Alessandro Bravetti[1,*,+] **and Pablo Padilla**[1,2,+]

[1]Instituto de Investigaciones en Matemáticas Aplicadas y en Sistemas, Universidad Nacional Autónoma de México, México City, 04510, México
[2]Fitzwilliam College, University of Cambridge, Storey's Way, CB3 ODG, UK
[*]alessandro.bravetti@iimas.unam.mx
[+]these authors contributed equally to this work

ABSTRACT

In this Supplementary Information we provide the necessary steps leading to the Optimal Replicator Equation (ORE), which is used in the main text to give a solution of the Prisoner's Dilemma.

Optimal Control Theory

Optimal Control Theory (OCT) is the mathematical framework suited for doing tasks in an "optimal" way, where the definition of optimality depends on the specific problem. For this reason it is widely used in a number of applications, such as engineering, biology, physics and economics[1–4]. Let us consider a dynamical system

$$\dot{\mathbf{x}} = \mathbf{F}(\mathbf{x},\mathbf{u}), \qquad \mathbf{x}(0) = \mathbf{x}_0, \tag{1}$$

where $\mathbf{x} = (x_1,\dots,x_n)$ are the variables representing the *state* of the system, while $\mathbf{u} = (u_1,\dots u_m)$ are the *control variables*, that is, variables whose evolution can be externally controlled. Typically one can control less variables than the ones representing the state of the system, so that $m \leq n$. Now let us suppose that there exists some measure of optimality, i.e. some quantity which we would like to maximize over some time interval τ. For instance, in economics this could be the total income after some time interval. Thus we are interested in maximizing a functional of the form

$$\mathscr{P}[\mathbf{u}(\cdot)] := \int_0^\tau L(\mathbf{x},\mathbf{u})\,\mathrm{d}t + g(\mathbf{x}(\tau)), \tag{2}$$

where $L(\mathbf{x},\mathbf{u})$ is a function of the current state of the system and of the current control (which in the example from economics would be the income at any specific time $0 \leq t \leq \tau$), and $g(\mathbf{x}(\tau))$ is a function depending only on the final state of the system (in the example this could be the dividends at the end of the time interval).

The basic problem of OCT is thus to find the optimal control strategy $\mathbf{u}^*(t)$ such that

$$\mathscr{P}[\mathbf{u}^*] = \max_{\mathbf{u}\in\mathscr{U}} \mathscr{P}[\mathbf{u}], \tag{3}$$

subject to the dynamical constraint (1) and for some given initial conditions $x_i(0) = x_i^0$. In (3) the set $\mathscr{U} \subset \mathbb{R}^m$ is the set of *admissible controls*. Indeed one can imagine that in practical situations the controls $\mathbf{u}$ cannot be chosen arbitrarily but they are constrained to be part of some (typically bounded) subset of $\mathbb{R}^m$. For instance, in economics we can consider the amount of production of a particular good as a control parameter. Obviously such parameter cannot be negative, nor infinite.

From (3) it should be clear in which sense the control is *optimal*: optimality is by definition the maximization of the functional $\mathscr{P}$.

One of the fundamental results in OCT is Pontryagin's Maximum Principle (PMP), which states that the optimal strategy $\mathbf{u}^*$ has to be found by introducing the *co-states* $\mathbf{p} = (p_1,\dots,p_n)$ and the *control Hamiltonian*

$$\mathscr{H}(\mathbf{x},\mathbf{p},\mathbf{u}) := \mathbf{p}\cdot\mathbf{F}(\mathbf{x},\mathbf{u}) + L \tag{4}$$

and then solving the corresponding system of equations

$$\dot{x}_a = \frac{\partial\mathscr{H}}{\partial p_a}, \qquad \dot{p}_a = -\frac{\partial\mathscr{H}}{\partial x_a}, \qquad a = 1,\dots,n, \tag{5}$$

together with the maximum condition:

$$\mathscr{H}(\mathbf{x}^*(t),\mathbf{p}^*(t),\mathbf{u}^*(t)) = \max_{\mathbf{u}\in U}\mathscr{H}(\mathbf{x}^*(t),\mathbf{p}^*(t),\mathbf{u}) \qquad \text{for} \quad 0\le t\le\tau\,, \tag{6}$$

the initial condition

$$\mathbf{x}^*(0) = \mathbf{x}_0 \tag{7}$$

and the terminal condition

$$p_a^*(\tau) = \frac{\partial g(\mathbf{x}^*(\tau))}{\partial x_a}\,. \tag{8}$$

The Optimal Replicator Equation

Now let us apply OCT to the RE (eq. (2) in the main text). For convenience, let us write the right hand side of the RE as $\mathbf{F}(\mathbf{x},\mathbf{f})$. This notation is useful in order to compare with the above description of OCT.

Usually in evolutionary dynamics the fitness vector $\mathbf{f} = (f_1,\dots,f_n)$ is assumed to be a preassigned function of $\mathbf{x}$. In the example of the PD we have $n = 2$ and $f_1 = f_C$ and $f_2 = f_D$ given as in eq. (3) in the main text. We know that the RE with the payoff structure as in (1) in the main text induces a dynamics which leads the defectors to take over the population. An alternative way to read this fact is that the dynamics leads to a minimization of the variance of the fitness of the population. In fact, the variance

$$\mathrm{var}(y) := \langle (y-\langle y\rangle)^2\rangle \tag{9}$$

of some quantity y is a measure of how spread is y outside of its average value $\langle y\rangle$. Clearly this quantity is greater than or equal to zero and it vanishes if and only if $y = \langle y\rangle$. Thus the variance of the fitness of a population is minimized by the process which leads all members of the population to have the same fitness, as it happens for the PD using the standard RE.

Noticing that we can write

$$\mathrm{var}(f) = \sum_{i=1}^n x_i(f_i-\langle f\rangle)^2 = \sum_{i=1}^n x_i(f_i-\langle f\rangle)(f_i-\langle f\rangle) = \sum_{i=1}^n \dot{x}_i(f_i-\langle f\rangle) = \sum_{i=1}^n \dot{x}_i f_i\,, \tag{10}$$

where in the third equality we have used the RE and in the last equality we have employed the conservation of probability $\sum_{i=1}^n \dot{x}_i = 0$, we recognize that for a large enough τ the dynamics induced by the standard RE in the case of the PD leads to a minimum of the following quantity

$$\mathrm{var}(f) = \sum_{i=1}^n \dot{x}_i f_i = \mathbf{f}\cdot\mathbf{F}(\mathbf{x},\mathbf{f})\,. \tag{11}$$

We remark however that such dynamical behavior is not related to an optimal control process in the case of the standard RE, because there are no free parameters to be optimized. In particular, the fitness values are known from the beginning and are expressed as in eq. (3) in the main text.

Here instead, in order to generalize the standard RE, we treat $\mathbf{f}$ as a control parameter and apply the general theory of optimal control presented above. To do so, we define the problem of maximizing the functional

$$\mathscr{P}[\mathbf{f}(\cdot)] := -\int_0^\tau \mathbf{f}\cdot\mathbf{F}(\mathbf{x},\mathbf{f})\,\mathrm{d}s + g(\mathbf{x}(\tau))\,, \tag{12}$$

where

$$g(\mathbf{x}(\tau)) := \langle \mathbf{f}(\mathbf{x}(\tau))\rangle \tag{13}$$

is the final average fitness of the population, subject to the dynamical constraint given by the RE. The rationale for the functional (12) is the following: the first term is the term corresponding to minimization of the variance of the fitness of the population (cf. eq. (11)), which, together with the dynamical constraint given by the RE, models the fact that evolution selects only one type of individuals within a population, i.e. those with higher fitness. The second term is a final reward depending on the average fitness of the population at the final time. This term accounts for the second aspect of our model, that is, the fact that at the final time $t = \tau$ a selection is assumed among populations themselves. Therefore only strategies with the best final

average population fitness survive (see also the discussion in the main text). In the Prisoner's Dilemma, as well as in examples from biology, the final reward is an important evolutionary advantage for each individual. Therefore we include it here and we show in the main text that the corresponding optimal dynamics leads directly to the emergence of cooperative behavior among selfish individuals.

By using the OCT to maximize (12) subject to the dynamical constraint given by the RE, we obtain the dynamical equations for the evolution of the frequencies $\mathbf{x}$ in the RE, together with the equations for the evolution of the fitnesses $\mathbf{f}$. In the following we return to the case $n = 2$ and derive explicitly such equations. To do so, we need to write down the full set of equations (5)–(8) stemming from to the optimization problem (12). First of all, the control Hamiltonian (4) in this case reads

$$\mathscr{H}(\mathbf{x},\mathbf{p},\mathbf{f}) = \sum_{a=1}^{2} (p_a - f_a)\, x_a\, (f_a - \langle \mathbf{f} \rangle)\,. \tag{14}$$

This Hamiltonian is quadratic in the controls $\mathbf{f}$ and therefore we can find an explicit solution to the maximum condition (6). We proceed as follows: we solve the conditions for an extremum of $\mathscr{H}$

$$\frac{\partial \mathscr{H}}{\partial f_a} = 0\,, \quad a = 1,2\,, \tag{15}$$

thus obtaining

$$f_a^* = \frac{1}{2} p_a^*\,, \quad a = 1,2\,. \tag{16}$$

Then by a direct substitution of f_a^* in $\mathscr{H}$ we obtain

$$\mathscr{H}_{\max}(\mathbf{x},\mathbf{p},\mathbf{f}^*) = \frac{1}{4} x_1 x_2 (p_1 - p_2)^2 \tag{17}$$

and we can verify that

$$\mathscr{H}_{\max}(\mathbf{x},\mathbf{p},\mathbf{f}^*) - \mathscr{H}(\mathbf{x},\mathbf{p},\mathbf{f}) = \frac{1}{4} x_1 x_2 (2f_1 - 2f_2 - p_1 + p_2)^2 \geq 0 \qquad \text{for all } x_1 \geq 0, x_2 \geq 0, p_1, p_2, f_1, f_2\,. \tag{18}$$

This proves that the values of $\mathbf{f}^*$ in (16) indeed make $\mathscr{H}$ attain its maximum possible value for any given choice of $\mathbf{x}$ and $\mathbf{p}$, so that condition (6) is satisfied by choosing $\mathbf{f}^*$ as in (16).

From (14) we can then compute the equations of motion (5), which, using (16) and after some algebra read

$$\dot{x}_a = \frac{x_a}{2} (p_a - \langle \mathbf{p} \rangle)\,, \tag{19}$$

$$\dot{p}_a = \frac{p_a}{2} \left(\langle \mathbf{p} \rangle - \frac{p_a}{2} \right)\,. \tag{20}$$

We call the system formed by eqs. (16), (19) and (20), together with the initial conditions (7) and the terminal conditions (8), the *Optimal Replicator Equation* (ORE) (cf. equations (5)–(8) in the main text).

References

1. Geering, Hans P. "Optimal control with engineering applications." Berlin Heidelberg (2007).
2. Lenhart, Suzanne, and John T. Workman. Optimal control applied to biological models. Crc Press, 2007.
3. Evans, Lawrence C. "An introduction to mathematical optimal control theory." Lecture Notes, University of California, Department of Mathematics, Berkeley (2005).
4. Fleming, Wendell H., and Raymond W. Rishel. Deterministic and stochastic optimal control. Vol. 1. Springer Science & Business Media, 2012.
